# Valorization of Goat Cheese Whey through an Integrated Process of Ultrafiltration and Nanofiltration

**DOI:** 10.3390/membranes11070477

**Published:** 2021-06-28

**Authors:** Antónia Macedo, David Azedo, Elizabeth Duarte, Carlos Pereira

**Affiliations:** 1 Polytechnic Institute of Beja, School of Agriculture, Rua Pedro Soares, Ap. 6158, 7801-908 Beja, Portugal; david_azedo@hotmail.com; 2 LEAF—Linking Landscape, Environment, Agriculture and Food, Instituto Superior de Agronomia, University of Lisbon, Tapada da Ajuda, 1349-017 Lisboa, Portugal; eduarte@isa.utl.pt; 3 Polytechnic Institute of Coimbra, School of Agriculture, 3045-601 Coimbra, Portugal; cpereira@esac.pt

**Keywords:** goat cheese whey, ultrafiltration, nanofiltration, dilution mode

## Abstract

Goat cheese whey is a co-product that comes from goat cheese manufacture. Due to its high organic load, adequate treatment is necessary before its disposal. Additionally, the recent growing interest in caprine products, attributed to their specific nutritional and nutraceutical characteristics, such as the lower allergenicity of their proteins and higher content of oligosaccharides, compared with bovine products, made the recovery of goat cheese whey a challenge. In this study, an integrated process for the recovery of sweet goat whey components was carried out. It includes filtration, centrifugation and pasteurization, followed by sequential membrane processes, ultrafiltration/dilution, nanofiltration of ultrafiltration permeates in dilution mode and the concentration/dilution of nanofiltration retentates. Ultrafiltration was performed with membranes of 10 and 1 kDa. Membranes of 10 kDa have higher permeate fluxes and, in a single stage of dilution, allowed for better protein retention and higher lactose purity, with a separation factor of 14. The concentration of lactose by nanofiltration/dilution led to the retention of almost all the lactose in retentates and to a final permeate, whose application in cheese dairy plants will allow for the total recovery of whey. The application of this integrated process in small- or medium-sized goat cheese dairies can represent an important contribution to their sustainability.

## 1. Introduction

Goat cheese whey is a liquid co-product of goat cheese manufacture. It retains about 55% of the nutrients found in milk, including lactose, soluble proteins, bioactive peptides, lipids, minerals and vitamins [1]. In comparison with bovine and sheep cheese whey, goat cheese whey contains a higher concentration of oligosaccharides, namely sialic acid, that seems to promote the development of infants’ brain [2]. Besides, it is rich in nonprotein nitrogen compounds, namely nucleotides and free amino acids, making it suitable for baby food or children with a cow’s milk allergy [3,4]. Therefore, there is new and growing interest in producing caprine products due to the nutraceutical and hypoallergenic properties of caprine milk compared to cow’s milk [5], which contributes to the increasing volumes of goat whey produced. Despite its nutritional and nutraceutical value, goat cheese whey is usually treated as waste, deposited in septic tanks or partially mixed with the wastewaters coming from cheese washing and the cleaning operations of equipment and from the cheese dairy; it is then delivered to wastewater plants. Its high values of chemical oxygen demand (COD) and biological oxygen demand (BOD_5_), about 50–120 g L^−1^ and 27–60 g L^−1^, respectively [6,7], can lead to the decline in treatment efficiency, transforming this co-product into one of the main environmental problems that the dairy industry has faced for decades. So, its reuse has the advantage of generating value-added products while mitigating its negative impacts on the environment.

Membrane technologies are the most common separation processes used for recovering valuable fractions from cheese whey. Ultrafiltration (UF) is well-established in the food industry to produce whey protein concentrates (WPC) from bovine cheese whey [8], using membranes with a cut-off equal to or higher than 10 kDa [9,10]. These membranes allow the retention of the predominant whey proteins (β-lactoglobulin (β-Lg), α-lactalbumin (α-La), immunoglobulin (Ig), serum albumin (SA)) and other minor proteins, such as lactoferrin (LF) and lactoperoxidase (LP), while lysozyme, glycomacropeptide (GMP) or bioactive peptides, with lower molecular weights or sizes [11], can mostly permeate through the membranes, together with lactose, other sugars and minerals. To increase the permeation of these smaller compounds, thus improving the separation of protein and lactose fractions, the dilution mode of UF retentates is also applied to obtain purified streams [12]. The recovery of permeates of ultrafiltration can be carried out by nanofiltration (NF), followed by the dilution mode of retentates [13]. NF, due to its specific characteristics, especially for the separation of the smaller solutes present in UF permeates, can be a valuable tool, retaining mostly lactose, the main component responsible for the environmental damage of whey [14]; bioactive peptides; oligosaccharides, free amino acids; and bivalent ions, originating a final permeate with a very low organic load [15,16]. The use of NF in dilution mode can enhance the permeation of salts, especially sodium and chloride, which can be high in UF permeates because of salt addition to milk during cheese manufacture. Therefore, the application of NF to UF permeates, in dilution mode, contributes to the reduction in osmotic pressure, a major drawback in these processes [17]. In addition, the purification of retentates may allow its application in food or pharmaceutical industries, due to their nutritional and nutraceutical characteristics, as cited above. This investigation aims to contribute to improvement in the sustainability of the artisanal production of goat cheese, carried out mainly in small- and medium-sized cheese dairy plants. This study has an innovative character in that it presents an integrated membrane process for the total recovery of a co-product with a very complex composition, such as goat cheese whey. Therefore, the main objectives are:

- to evaluate the performance of UF membranes of different cut-offs in the separation of the protein and lactose fractions of goat cheese whey;
- to investigate the influence of the dilution mode, in three stages, applied to UF retentates in separation efficiency;
- to assess the performance of the concentration process of nanofiltration, in dilution mode, of UF permeates;
- to study the influence of dilution, in three stages, applied to NF retentates for the removal of salts and to purify lactose;
- to produce a permeate with a low organic content.

## 2. Materials and Methods 

### 2.1. Sampling and Pretreatment of Goat Cheese Whey

Six samples of goat cheese whey (GCW) were collected in the same cheese dairy, located close to Beja, Portugal. The GCW was produced during the cheese-making process, which involved the following steps: milk pasteurization; filtration of the milk; addition of salt to the milk cheese; rennet coagulation; syneresis, where whey was released; the new addition of salt to the curd; pressing and packing, before expedition. A volume of about 10 L of each sample was collected and carried out to our laboratory, keeping them refrigerated in ice during transportation. After arriving, samples were filtrated two times through cotton cloths, like those used in a traditional cheese dairy, to remove suspended solids and casein fines. After that, samples were skimmed in an Elecrem SAS, Fresnes, France, centrifuge, at a temperature of about 35 °C to remove most of the lipids and some minor residues of casein and bacteria. Since these samples have a high concentration of lipids, the reduction of their concentration is crucial to avoid membrane fouling. Finally, whey samples were subjected to a low pasteurization process, at 65 °C, for 30 min. When it was not possible to process the samples in the same day, they were immediately preserved at about 3 °C until the next day.

### 2.2. Permeation Experiments

All the permeation experiments were carried out in a plane-and-frame module, Lab Unit M20, from Alfa Laval, Navskov, Denmark. This filtration rig is a commercial installation with a membrane area ranging from a minimum of 0.036 m^2^, which corresponds to two membrane sheets, to 0.72 m^2^, the maximum area. The membranes were grouped in pairs, resting on the top and bottom of the same support plate. The plates were separated by spacers that acted as feed chambers 0.5 mm in height and that were divided into 30 channels to increase tangential velocity and thus, promote mass transfer in the adjacent layer near the membrane surface. In each support plate, there were individual collectors for the permeate. Support plates and spacers were made of polysulfone, and the module frame was made of stainless steel. The unit had a hydraulic system that allowed the flat plate module to be compressed, making it perfectly watertight.

**Figure 1:**
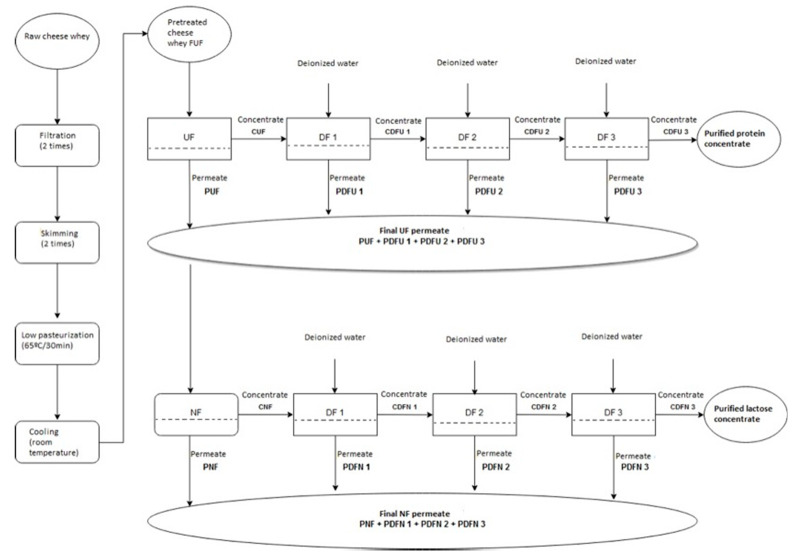
Experimental scheme.

These experiments included the UF of the pretreated goat cheese whey to remove residual protein and fat and to obtain a protein-containing retentate and a lactose-rich permeate. For a better recovery of lactose in permeates, dilution mode in ultrafiltration (DUF) of the retentates was also carried out. After that, all of the permeates resulting from UF and DUF were mixed and subjected to NF to recover the lactose fraction. For purifying this lactose-rich retentate, which may increase the possibilities for its use in various industries, dilution mode in NF was also applied to the retentates of the NF process (Figure 1). 

Before each permeation test, the hydraulic permeability to pure water was determined by measuring the permeate fluxes at different transmembrane pressures at a feed circulation velocity of 0.94 ms^−1^ and using Equation (1). The hydraulic permeability of pure water is the slope of the linear regression obtained from the experimental water fluxes and corresponding transmembrane pressures.

(1) $$J_{w}=\left(\frac{L_{p}}{\mu }\right)\Delta P$$

where $J_{w}$ is the water permeate flux (ms^−1^); $\left(L_{p}/\mu \right)$ is the hydraulic permeability to pure water (ms^−1^Pa^−1^); $L_{p}$ is the intrinsic permeability of the membrane (m), related with its morphological characteristics; μ is the water viscosity (Pa·s), and ΔP is the applied transmembrane pressure (Pa).

After the tests, a cleaning and disinfection cycle was performed, according to the procedure shown in Table 1. To ensure that membrane’s permeability characteristics were kept, the hydraulic permeability to pure water was again determined and, if it was at least 95% of the initial value, the same membranes were used in the following tests. 

#### 2.2.1. Ultrafiltration Experiments

Ultrafiltration experiments were carried out with two different kinds of membranes, one with an active layer made of regenerated cellulose acetate and a molecular weight cut-off of 10 kDa, designated as RC70PP, and another one with an active layer made of a composite fluoropolymer and a molecular weight cut-off of 1 kDa, named ETNA01PP. Both materials used to manufacture the membranes are hydrophilic in nature, which minimizes the effects of fouling by organic matter, particularly proteins, as described elsewhere [18]. The best operating conditions of transmembrane pressure (0.2 MPa) and feed circulation velocity (0.94 ms^−1^) were selected, based on the results obtained in total recirculation UF experiments, carried out in the range of transmembrane pressure between 0.1–0.4 MPa, with the same membranes used in this study and established in previous works [18,19]. The highest permeate flux and relative flux (*J_p_*/*J_w_*) and the best separation between protein and lactose fractions were the criteria used for selection. The temperature varied from 16 °C to 22 °C and, to correct for the different viscosities of the permeates, all the permeate fluxes were converted to 25 °C [20]. The pressure drop along the module was about 0.1 MPa.

The first set of ultrafiltration experiments was done in three steps: preconcentration until the volume concentration factor, VFC = 2.0; dilution mode by adding deionized water; and postconcentration. This procedure allows the achievement of the concentration/purification of the protein fraction in the retentate and a better recovery of lactose in the permeate, thus contributing to improvements in the separation of these components.

Starting from an initial volume of 8.75 L of each sample, three UF experiments with GCW were performed in concentration mode until a volume concentration factor (VCF) of about two was reached with each of the membranes (10 kDa and 1 kDa), using a membrane area of 0.072 m^2^. After concentration, the dilution (DF) of the final retentates was realized in three stages in a discontinuous mode. In each of them, a volume of deionized water, equal to the observed volume of the retentate in the tank, was added. After homogenization and stabilization at the same operating conditions of transmembrane pressure, feed circulation velocity and temperature, a new concentration process took place until the same volume of permeate was collected, thus maintaining the volume of the retentate. Samples of raw and pretreated GCW, retentates and permeates of UF and retentates and permeates of DF were taken for analyses.

The results obtained from this first set of experiments, with both membranes, were analyzed in terms of the following parameters: productivity, measured by volumetric permeate fluxes (*J_p_*) and their evolution along the concentration processes, and the separation factor, α, for lactose and protein, which should be greater than 1; the higher it is, the better the separation between the two solutes.

The volumetric permeate fluxes were determined according to Equation (2):

(2) $$J_{p}=\frac{\Delta V}{A_{m}\times \Delta t}\;\;\;\;$$

where $J_{p}$ is the volumetric permeate flux; ΔV is the volume (m^3^) of permeate collected during an interval of time Δt (s), and $A_{m}$ is the total membrane area (m^2^).

The separation factor, α, is defined as [21]:

(3) $$\alpha =\frac{S_{microsolute}}{S_{macrosolute}}$$

where $S_{microsolute}$ is the sieving coefficient for the microsolute (lactose), and $S_{macrosolute}$ is the sieving coefficient for the macrosolute (protein). $S_{i}$, the sieving coefficient for a component *i*, is given by: *S_i_* = *c_p_*/*c_r_*, in which *c_r_* is the concentration of a solute in the bulk retentate, and *c_p_*, the concentration of the solute in the bulk permeate. 

Based on the results obtained in this first set of experiments, the best membrane in terms of productivity and the separation of protein and lactose fractions was selected to carry out NF experiments.

#### 2.2.2. Nanofiltration Experiments

Nanofiltration experiments were realized with the mixture of permeates resulting from the UF (PUF+PDFU1+PDFU2+PDFU3) experiments (Figure 1). Permeates were mixed, homogenized and subjected to NF. NF experiments were performed with membrane NFT50 (NF), commercialized by Alfa Laval, Navskov, Denmark. These membranes have an active layer made of polyamide semi-aromatic (polipiperazine). The preconcentration of the feed was carried out until a VCF of about 2.0, at a transmembrane pressure of 2 MPa, a feed circulation velocity of 0.94 ms^−1^ and a membrane area of 0.072 m^2^. The diafiltration of the final retentates of NF was performed in three stages by adding a volume of deionized water equal to that of the retentate in the tank, and, afterwards, the concentration process proceeded until an identical volume of permeate was collected. The experimental conditions used in this process were the same as those used for preconcentration by NF. 

The performance of the process of concentration by NF, in dilution mode, followed by the diafiltration of the retentates obtained, was determined in terms of permeate fluxes (productivity), *J_p_*; efficiency of the removal of salts, $\mu_{removal}$; and the evaluation of the quality of the final permeate for possible further application in cheese dairy plants.

The efficiency of removal of a certain solute is given by:

(4) $$\mu_{removal}=\frac{c_{ri}-c_{rf}}{c_{ri}}\times 100$$

where *c_ri_* is the concentration of a solute in a retentate *i*, before a stage of diafiltration, and *c_rf_* is its concentration in the retentate after that stage.

### 2.3. Cleaning and Disinfection Cycle

After the permeation experiments, samples were removed from the installation, and three flushes were carried out with water to ensure that no residues were present. The cleaning and disinfection cycle realized and the operating recommended conditions of the manufacturer for the membranes in this study are shown in Table 1. The cleaning procedure included four steps, in each one a different chemical was added, performed under recirculating conditions, which means that both permeate and retentate were recycled to the feed/retentate tank. A transmembrane pressure of 0.1 MPa, a feed circulation velocity of 0.92 ms^−1^ and a temperature of 25 °C were used during this operation. For NF membranes, a transmembrane pressure of 1 MPa was applied, maintaining the same values of feed circulation and temperature. Between each two cleaning solutions, water was permeated to remove the previous reagent, checking if the pH was already restored. After cleaning, a final disinfection step was carried out, as presented in Table 1, using the same transmembrane pressure, feed circulation velocity and temperature.

### 2.4. Physicochemical Characterization of the Samples

The samples (feed, retentates and permeates) were analyzed for: pH (by potentiometry); lactose, by determination of reducing sugars [22]; total solids, by gravimetry [23]; total nitrogen, by the Kjeldahl reference method; crude protein, obtained from total nitrogen multiplied by the factor 6.38 [24] and adapted for cheese whey; the fat content, determined by infrared spectroscopy using the equipment Milkoscan134B, previously calibrated for cheese whey with the standard method of Rose-Gottlied for milk and dairy products; sodium and potassium, by emission flame photometry, according to the procedure described in [25]; calcium and magnesium by atomic absorption spectrophotometry with air–acetylene flame [25]; chloride, by volumetric precipitation, according to the method of Charpentier-Volhard [26]; and phosphates, by the spectrophotometric method of ammonium molybdate [27]. 

## 3. Results and Discussion

### 3.1. Physicochemical Characterization of Raw and Pretreated Goat Cheese Whey

The average composition of raw and pretreated goat cheese whey is shown in Table 2.

The goat cheese whey used in this study is classified as a sweet cheese whey because its pH is around 6.0 and is produced from milk coagulated by the enzymatic hydrolysis of casein through chymosin action, at a pH not lower than 5.6 [28]. Apart from water (around 90.8% *w*/*w*), the main components are lactose, followed by minerals, lipids and nitrogen compounds. Lactose, lipids, and nitrogen compounds are, in order of importance, primarily responsible for the high organic loading of these co-products, which are translated into high levels of COD and BOD, as stated in Section 1.

The pretreatment realized (filtration, centrifugation, pasteurization) allowed for a removal of about 58% of lipids, 5% of nitrogen compounds and 15% of ash, leading to a decrease of 6% of the total solids. These results suggest that a part of the organic matter present in the raw goat cheese whey, mainly related to its lipid content, was quickly removed during the pretreatment. This co-product, rich in lipids, after pasteurization, may eventually be reused in cheese dairies, added to milk cream to increase the yield of the manufacture process of goat butter and/or other types of spreads, and will be the subject of further study. However, lactose, most of the nitrogen fraction and around 42% of fat is still present in the pretreated goat cheese whey, thus contributing to its high content of organic matter.

Regarding the mineral composition, the most salient aspect is the very high concentrations of chloride and sodium, which are in contrast with goat milk, where the dominant minerals are potassium, chloride, calcium and phosphate [4]. This resulted from the addition of sodium chloride to the cheese milk, during the manufacture of goat cheese. 

### 3.2. Permeation Experiments

#### 3.2.1. Characteristics of Membranes

Before permeation experiments, the hydraulic permeability of membranes to pure water was determined (Table 3), according to the procedure described in Section 2.2. In Table 3 is displayed the hydraulic permeability of membranes, the intrinsic permeability and the MWCO of membranes, furnished by the supplier and determined, for NF membranes, according to the procedure described elsewhere [16].

#### 3.2.2. Performance of Ultrafiltration Experiments

- Permeate fluxes

**Figure 2:**
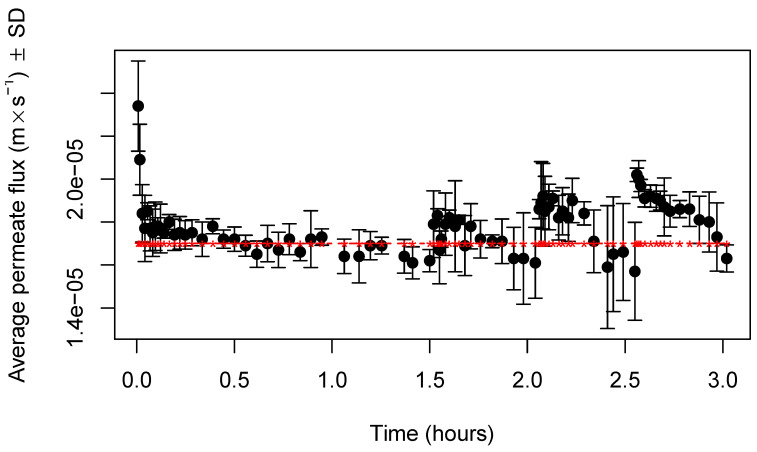
Variation of average permeate fluxes (±standard deviation) with time, during the process of UF/DF, obtained with membranes RC70PP (n = 3 experiments), at a transmembrane pressure of 0.2 MPa.

**Figure 3:**
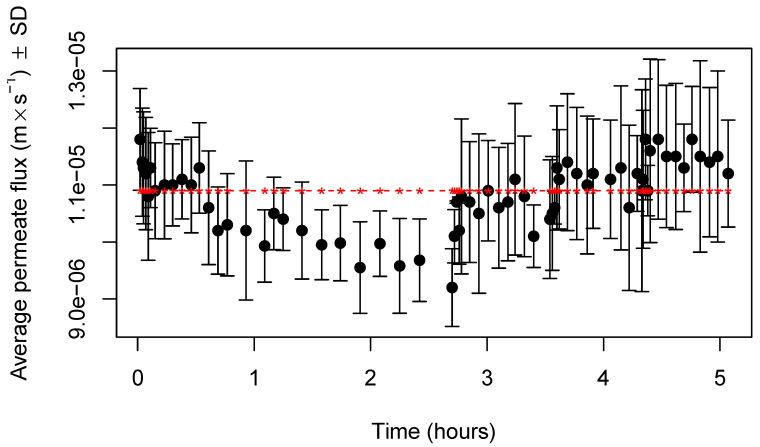
Variation of permeate fluxes (±standard deviation) with time, during the process of UF/DF, obtained with membranes ETNA01PP (n = 3 experiments), at a transmembrane pressure of 0.2 MPa.

The evolution of permeate fluxes along the process of concentration by UF, followed by dilution in UF mode with three stages for both types of membranes (RC70PP and ETNA01PP), is displayed in Figure 2 and Figure 3, respectively. The horizontal line, in both figures, represents the water fluxes at the transmembrane pressure of 0.2 MPa, at which permeation experiments were carried out. As can be observed, until a VCF of about 2.5, the permeate fluxes obtained with samples and with water are close, which indicates that, in the experimental conditions used, fouling is negligible for both membranes. More experiments will be realized in the future on the highest VCFs to study flux behavior when protein concentration is increased. The effect of MWCO, and the corresponding mean pore radius, is evident, because permeate fluxes obtained with membranes of 1 kDa were around 50% of those produced with membranes of 10 kDa. Therefore, the use of membranes with higher MWCO allowed for higher permeate fluxes, as expected, probably because both membranes are made from hydrophilic materials, which are less susceptible to fouling by proteins, the component most responsible for this phenomena in the UF of cheese whey.

In the case of UF experiments with the membrane RC70PP, at the very beginning, a decline in permeate fluxes was observed, probably due to polarization-concentration phenomena, which is more important when permeate fluxes are higher, due to the rapid accumulation of retained compounds near the membrane surface. However, after that, an average constant flux of about $1.73\times 10^{-5}$ ms^−1^ was reached. With membranes ETNA01PP (Figure 3), the initial decline in permeate fluxes was much less pronounced, because the lower average permeate flux, around $1.05\times 10^{-5}$ ms^−1^, minimized the effect of the intensity of concentration-polarization phenomena [29]. 

Relative to the DF process carried out in three stages, we can observe in Figure 2 that the intensity of average permeate fluxes along the dilution processes were slightly higher than those observed during preconcentration by UF, because are the lower species, such lactose and minerals that are preferentially permeating UF membranes. During each stage, permeate fluxes were kept almost constant. The range of permeate fluxes during the dilution process realized with membranes RC70PP was between $1.79\times 10^{-5}$ ms^−1^ for DF1 and $2.00\times 10^{-5}$ ms^−1^ at the third stage (DF3). The same was true for the dilution process performed with membranes ETNA01PP (Figure 3), the permeate fluxes ranging from $1.08\times 10^{-5}$ ms^−1^ (DF1) up to $1.17\times 10^{-5}$ ms^−1^ (DF3). These results show that permeate fluxes, during the dilution processes, were not affected by the permeation of the lower species across UF membranes.

- Analysis of separation factors

The concentrations of lactose and protein in retentates and permeates, as well as the corresponding separation factors between these two components, along UF/DF processes, are shown in Table 4 and Table 5 for membranes RC70PP and ETNA01PP, respectively.

The observation of the data displayed in Table 4 allows the conclusion that the use of dilution in ultrafiltration mode for UF retentates led to a large increase in the separation factor between lactose and protein, from 6 to 14, right after the first stage of dilution. This is mainly due to the permeation of lactose into permeate streams, as can be confirmed by the decrease in its concentration in UF retentates. However, after the first stage (DF1), the separation factor between those components remained or even declined. Therefore, since dilution mode involves water consumption, which should be minimized for economic and environmental reasons, the use of a second and third stage in dilution mode will be dependent on the desired purification of the final protein fraction.

For preconcentration by UF, the membranes of lower MWCO (1 kDa) led to a better separation between lactose and protein, because α is around 10 and, for the other membranes, it is 6 (Table 5). This is likely due to the greater accumulation of the protein fraction in retentates and, consequently, the lower loss of protein into the permeates, probably the lower-molecular-weight whey proteins, such as glycomacropeptide (GMP), with a molecular weight of 6.80 kDa, and bioactive peptides, as described elsewhere [9]. However, unlike what was observed with membranes of 10 kDa, during the dilution process in three stages, the separation factor decreased to about 9 and was kept constant until the end of this process. This may be due to the higher accumulation of the protein fraction in retentates that may have hampered the removal of lactose into the permeate stream, leaving it retained within the protein fraction, which can be confirmed by its higher concentration in retentates. Therefore, relative to the separation factor, despite the fact that membranes of lower MWCO allowed, in a single UF operation, the obtention of a better separation between protein and lactose fractions, the final decision as to which of the membranes should be selected will depend on the intended application of protein retentates.

#### 3.2.3. Performance of Nanofiltration Experiments

- Variation of permeate fluxes with VCF

Nanofiltration experiments were carried out with the permeates resulting from the UF/DF of membranes with the higher MWCO because their separation between the protein and lactose fractions was better. During the process of dilution in the nanofiltration mode of UF permeates, it was observed a sharp decline in the average permeate flux of about 45%, ranging from $1.59\times 10^{-5}$ ms^−1^ (57.24 Lh^−1^m^−2^) to $8.33\times 10^{-6}$ ms^−1^ (29.99 Lh^−1^m^−2^), and until the VCF of 2.34 was attained. A similar pattern was observed during the DF of the NF retentates performed in three stages. Permeate fluxes were only slightly higher than those measured during the preconcentration process by NF, ranging from $1.74\times 10^{-5}$ to $8.72\times 10^{-6}$ ms^−1^, and the decline of permeate fluxes varied from 39 to 47%. This behavior is explained by the fact that UF permeates are mainly composed of the smaller solutes of cheese whey, like lactose and minerals, especially sodium, chloride and potassium, that mostly contribute to its higher osmotic pressure [11], leading to a decrease in effective membrane pressure and thus, to the decrease of permeate fluxes. One possibility to overcome this disadvantage will be the reduction of the amount of sodium chloride added to the milk and curd during the manufacturing process, which will also be beneficial for human health. Another factor that can also contribute to the decrease in NF productivity is the possible formation of insoluble salts of calcium or magnesium phosphates near the membrane surface due to their high retention by NF membranes.

- Physicochemical characterization of NF and DF/NF samples and removal efficiency

Table 6 shows the physicochemical characterization of the following samples (Figure 1): final UF permeate, which is the feed for nanofiltration; CNF, the concentrate of nanofiltration; PNF, the permeate of nanofiltration; CDNF3, the concentrates of 3rd stage of diafiltration; and PDNF3, the corresponding permeates.

In Table 6, it can be observed that the main components of the feed of the DF/NF process (mixture of all the permeates from ultrafiltration) are, apart from water, lactose and minerals, nitrogen compounds at the lowest concentration (0.023% *w*/*w*). During the previous ultrafiltration, most nitrogen compounds were retained by UF membranes, in accord with the sieving coefficient of about 8% (Table 4), which corresponds to a membrane rejection around 92%. 

Lipids and nitrogen compounds were preferentially retained by NF membranes, probably through steric hindrance and non-electrostatic membrane–solute interactions, the main mechanisms responsible for the retention of uncharged molecules in nanofiltration membranes [30,31]. 

Relative to minerals, chloride and sodium are predominant due to their preferential permeation through UF membranes, as expected when negligible fouling problems occur. The distribution of ions between retentates and permeates in nanofiltration can result both from steric hindrance and electrostatic interactions between ions and surface charge, based on the Donnan exclusion mechanism [31]. Since NF membranes used in this work have an isoelectric point, pH_i_ = 4.2, this means that, at the pH of our samples, they carried a negative charge [30]. Then, the counter-ions, especially calcium and magnesium due to their higher density charge, were adsorbed at the membrane surface by electrostatic interactions, and the co-ions, such as chloride, were mainly repulsed by the membrane surface to satisfy the electroneutrality condition. The chloride ion even had a negative rejection during the concentration process by NF, probably because of its higher density charge. 

In NF/DF stages, the concentration of monovalent ions clearly decreased due to their removal into the permeate streams. The removal efficiencies of salts were calculated based on their concentrations in CNF and CDNF3 (Table 6) and using Equation (4). Calcium and magnesium were preferentially retained (with negative removals); phosphates were slightly removed (circa 2.5%); potassium, sodium and chloride had removal efficiencies of 44%, 54% and 78%, respectively, after the three-stage DF process. It is possible that the high retention of calcium and magnesium contributed to a small reduction in the surface charge of the membrane, and therefore, to a lower removal of the chloride anion present in the retentates.

The predominant components of NF permeate are water, chloride and sodium, and thus, they can be used in cheese dairy plants as washing waters for cheese or during cheese processing. 

## 4. Conclusions

The fractionation of goat cheese whey using the sequential membrane processes proposed in this study allowed a good separation between protein and lactose fractions through ultrafiltration followed by diafiltration. The recovery of lactose by the nanofiltration of permeates contributes to minimizing the environmental impact of this co-product of goat cheese manufacture and, at the same time, allows for possible applications of the separated fractions. 

## Figures and Tables

**Table 1:** Process of cleaning and disinfection of membranes ^a^.

		Membranes		Time (min)
Membrane parameters	RC70PP (UF)	ETNA01PP (UF)	NF	
pH	1–10	1–11	2–11	
Transmembrane pressure (MPa)	0.1–0.5	0.1–0.5	0.1–1	
Temperature (°C)	0–60	0–65	0–45	
**Cleaning**				
NaOH (% *w*/*v*)	0.05	0.05	0.05	15
Na-EDTA (% *w*/*v*)	0.20	0.20	0.20	15
HNO_3_ (% *w*/*v*)	0.25	0.25	0.10	15
Citric acid (%*w*/*v*)	0.50	0.50	0.50	15
**Disinfection**				
H_2_O_2_ (mgL^−1^) at 25 °C	1000	1000	1000	30

^a^ Into tolerate pH limits of membranes; Na-EDTA, ethylenediaminetetra-acetic acid, sodium salt.

**Table 2:** Average composition (mean ± standard deviation) of raw goat cheese whey and pretreated goat cheese whey ^a^.

Parameters	Raw Goat Cheese Whey	Pretreated Goat Cheese Whey (FUF)
pH (25 °C)	5.68 ± 0.72	5.90 ± 0.79
Total solids (% *w*/*w*)	9.24 ± 0.50	8.68 ± 0.47
Lactose (%*w*/*w*)	5.11 ± 0.33	5.12 ± 0.21
Lipids (% *w*/*w*)	1.06 ± 0.19	0.44 ± 0.09
Nkjeldahl (% *w*/*w*)	0.082 ± 0.005	0.078 ± 0.006
Crude protein (% *w*/*w*)	0.53 ± 0.03	0.50 ± 0.04
Ash (% *w*/*w*)	2.13 ± 0.03	1.81 ± 0.11
Cl (mg L^−1^)	9553 ± 217	8984 ± 336
P (mg L^−1^)	501 ± 98	392 ± 31
Ca (mg L^−1^)	171 ± 0.5	161 ± 0.3
Mg (mg L^−1^)	92.0 ± 0.3	74.4 ± 0.3
K (mg L^−1^)	1875 ± 3.4	1632 ± 7.6
Na (mg L^−1^)	6574 ± 235	5730 ± 186

^a^ n (number of samples) = 6.

**Table 3:** Hydraulic permeability of membranes (±95% confidence interval), intrinsic permeability and MWCO of membranes.

Membrane	$\mathrm{Hydraulic}\;\mathrm{Permeability}\;\mathrm{to}\;\mathrm{Pure}\;\mathrm{Water}\;\left(L_{p}/\mu \right)$ (ms^−1^Pa^−1^)	$\mathrm{Intrinsic}\;\mathrm{Permeability}\;L_{p}(m)\;{}^{\left(1\right)}$	**MWCO** **kDa**
RC70PP (UF)	$1.73\times 10^{-10}\pm 2.83\times 10^{-11}$	$1.54\times 10^{-13}$	10
ETNA01PP (UF)	$4.85\times 10^{-11}\pm 3.84\times 10^{-13}$	$4.32\times 10^{-14}$	1
NFT50 (NF)	$1.48\times 10^{-11}\pm 4.56\times 10^{-13}$	$1.32\times 10^{-14}$	0.13 ^(2)^

^(1)^ The dynamic viscosity of pure water, at a temperature of 25 °C, used to calculate the intrinsic permeability of membranes, was $8.91\times 10^{-4}$ Pa·s; ^(2)^ In accord with [16], for the same set of membranes.

**Table 4:** Separation factors ^(1)^ (α) between lactose and protein, along with UF/DF processes for membrane RC70PP.

Processes	C_p_ (Lac) (% *w/w*)	C_r_ (Lac) (% *w/w*)	C_p_ (prot) (% *w/w*)	C_r_ (prot) (% *w/w*)	α
UF	5.27 ± 0.68	5.31 ± 0.21	0.14 ± 0.06	0.84 ± 0.04	6.0 ± 0.4
DF1	3.42 ± 0.25	3.53 ± 0.26	0.06 ± 0.04	0.89 ± 0.16	14.4 ± 0.6
DF2	2.08 ± 0.37	2.24 ± 0.42	0.06 ± 0.05	0.93 ± 0.04	14.4 ± 0.5
DF3	1.26 ± 0.33	1.52 ± 0.45	0.10 ± 0.02	1.26 ± 0.11	10.4 ± 0.4

^(1)^ α was determined at a transmembrane pressure of 0.2 MPa, feed circulation velocity of 0.92 ms^−1^ and temperature of 25 °C.

**Table 5:** Separation factors ^(1)^ (α) between lactose and protein, along with UF/DF processes for membrane ETNA01PP.

Processes	C_p_ (Lac) (% *w/w*)	C_r_ (Lac) (% *w/w*)	C_p_ (prot) (% *w/w*)	C_r_ (prot) (% *w/w*)	α
UF	5.08 ± 0.13	5.91 ± 0.08	0.09 ± 0.03	1.05 ± 0.05	10.0 ± 0.1
DF1	3.20 ± 0.11	3.83 ± 0.01	0.11 ± 0.02	1.10 ± 0.04	8.4 ± 0.2
DF2	2.26 ± 0.01	2.77 ± 0.07	0.10 ± 0.02	1.08 ± 0.05	8.8 ± 0.3
DF3	1.52 ± 0.07	2.11 ± 0.07	0.09 ± 0.01	1.08 ± 0.05	8.6 ± 0.2

^(1)^α was determined at a transmembrane pressure of 0.2 MPa, feed circulation velocity of 0.92 ms^−1^ and temperature of 25 °C.

**Table 6:** Physicochemical characterization of NF and DF/NF samples.

Parameters	Feed	CNF	PNF	CDNF3	PDNF3
pH (25 °C)	6.28 ± 0.02	6.27 ± 0.01	6.20 ± 0.30	6.33 ± 0.05	5.99 ± 0.09
Total solids (%*w*/*w*)	6.93 ± 0.39	12.49 ± 0.04	1.68 ± 0.79	12.74 ± 0.87	1.16 ± 0.04
Lactose (%*w*/*w*)	4.67 ± 0.10	9.49 ± 0.46	n.d. ^(1)^	10.01 ± 0.14	n.d.
Lipids (%*w*/*w*)	0.043 ± 0.01	0.057 ± 0.02	0.046 ± 0.05	0.070 ± 0.01	0.061 ± 0.02
N_Kjeldahl_ (%*w*/*w*)	0.023 ± 0.003	0.034 ± 0.004	0.021 ± 0.007	0.042 ± 0.002	0.010 ± 0.002
Crude protein (%*w*/*w*)	0.15 ± 0.02	0.22 ± 0.02	0.13 ± 0.03	0.27 ± 0.05	0.05 ± 0.02
Ash (%*w*/*w*)	1.29 ± 0.21	2.23 ± 0.25	1.59 ± 0.72	0.89 ± 0.04	0.25 ± 0.02
Cl (mg L^−1^)	9351.3 ± 961.1	12,338.1 ± 1760.0	12,835.8 ± 754.3	2702.3 ± 497.8	2015.0 ± 249.7
P (mg L^−1^)	203.23 ± 26.32	215.33 ± 16.82	n.d.	209.93 ± 24.25	n.d.
Ca (mg L^−1^)	112.80 ± 0.18	163.20 ± 0.51	n.d.	168.40 ± 0.35	n.d.
Mg (mg L^−1^)	87.20 ± 0.17	128.00 ± 0.38	n.d.	146.60 ± 0.44	n.d.
K (mg L^−1^)	163.20 ± 0.18	138.89 ± 0.25	126.74 ± 0.14	78.13 ± 0.12	53.82 ± 0.11
Na (mg L^−1^)	5447.89 ± 234.13	4240.64 ± 265.12	4200.40 ± 186.71	1946.88 ± 256.21	699.40 ± 38.24

^(1)^ Not detectable.

## Data Availability

All the data of this study are presented along the text.
